# Extensive Frontoparietal Abscess: Complication of Frontal Sinusitis (Pott's Puffy Tumor)

**DOI:** 10.1155/2014/632464

**Published:** 2014-05-25

**Authors:** Raquel Andrade Lauria, Fernando Laffitte Fernandes, Thiago Pires Brito, Pablo Soares Gomes Pereira, Carlos Takahiro Chone

**Affiliations:** Department of Otolaryngology, Head and Neck, University of Campinas (UNICAMP), 251 Vital Brasil Street, 13083-888 Campinas, SP, Brazil

## Abstract

First described in 1768, the Pott's puffy tumor is a subperiosteal abscess associated with frontal bone osteomyelitis, resulting from trauma or frontal sinusitis. The classic clinical presentation consists of purulent rhinorrhea, fever, headache, and frontal swelling. The diagnosis is confirmed by CT scan and treatment requires intravenous antibiotics, analgesia, and surgical intervention. Early diagnosis and aggressive medical and surgical approach are essential for a good outcome. It rare and the early diagnosis is important; we describe the case of a 14-year-old adolescent with Pott's puffy tumor who was initially treated inadequately, evolving with extensive frontoparietal abscess. The patient underwent surgical treatment with endoscopic endonasal and external approaches combined. Intravenous antibiotics were prescribed for a prolonged time, with good outcome and remission of the complaints.

## 1. Introduction

The sinusitis suppurative complications are rare events but have high morbidity and mortality. Frontal bone osteomyelitis associated with subperiosteal abscess, also known as Pott's puffy tumor, is an example of such complications and can occur due to trauma or frontal sinusitis. First described by Percival Pott in 1768, it is a rare clinical entity, especially after antibiotics development. It affects most frequently the pediatric and immunologically compromised patients [1–3].

Patients classically present with complaints of purulent rhinorrhea, frontal headache, fever, vomiting, and frontal swelling [2, 3]. Computed tomography scan is an important exam to diagnose and assess associated complications and extension of nasal sinuses involvement. The treatment is imminently surgical and should be associated with prolonged antibiotic therapy, preferably guided by culture.

Here we report the case of an adolescent with extensive frontoparietal abscess secondary to frontal sinusitis, with good outcome after treatment.

## 2. Case Report

A 14-year-old male adolescent, with no significant past medical history, presented at an otorhinolaryngology department of a tertiary teaching hospital with a 7-day history of a common cold that progressively evolved with frontal headache, vomiting, fever, purulent rhinorrhea, and frontal and periorbital swelling. He had been treated in another service where he remained hospitalized for eight days, being started on amoxicillin-clavulanate 3 g/day for seven days with partial improvement of periorbital edema. He maintained, however, daily fever and progressive worsening of the frontal swelling.

**Figure 1:**
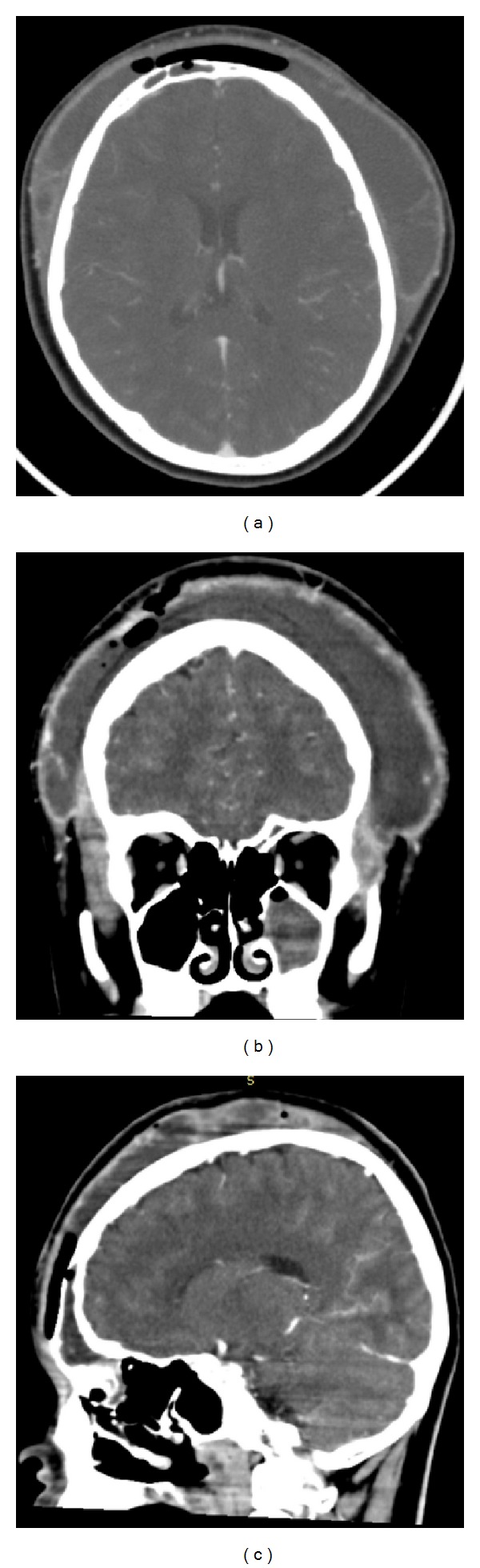
Axial (a), coronal (b), and sagittal (c) CT without contrast demonstrating soft tissue swelling of the forehead and parietal region; erosion of anterior table of the frontal sinus; frontal, maxillary, and anterior ethmoid sinuses opacification; subcutaneous emphysema.

Physical examination on admission revealed extensive painful and fluctuant swelling of the forehead and parietal region, periorbital edema, and spontaneous purulent lacrimal discharge (Figure 1(a)). Anterior rhinoscopy showed a hyperemic mucosa, nonobstructive turbinates, and mucopurulent discharge in common meatus bilaterally. Nasofibroscopy showed bilateral frontal recess purulent discharge, greater on the left. It showed no neurological abnormalities or signs of systemic involvement. The remainder of the physical exam was unremarkable.

Patient underwent CT scan of the paranasal sinuses which showed the following changes: erosion of anterior table of the frontal sinus, frontal, maxillary, and anterior ethmoid sinuses opacification, subcutaneous emphysema, and soft tissue swelling of the forehead and parietal region.

Due to the abscess extension, the patient was opted for surgical drainage with combined approach (endoscopic endonasal and external drainage via coronal incision), with drainage of approximately five hundred milliliters of thick purulent fluid sent for culture (Figure 3(a)). Intraoperative inspection showed frontal and temporal muscles necrosis, associated with osteomyelitis and erosion of the external table of the frontal bone. Additionally, endoscopic intranasal frontal sinusotomy was performed. (Figures 3(b), 3(c), and 3(d)).

**Figure 2:**
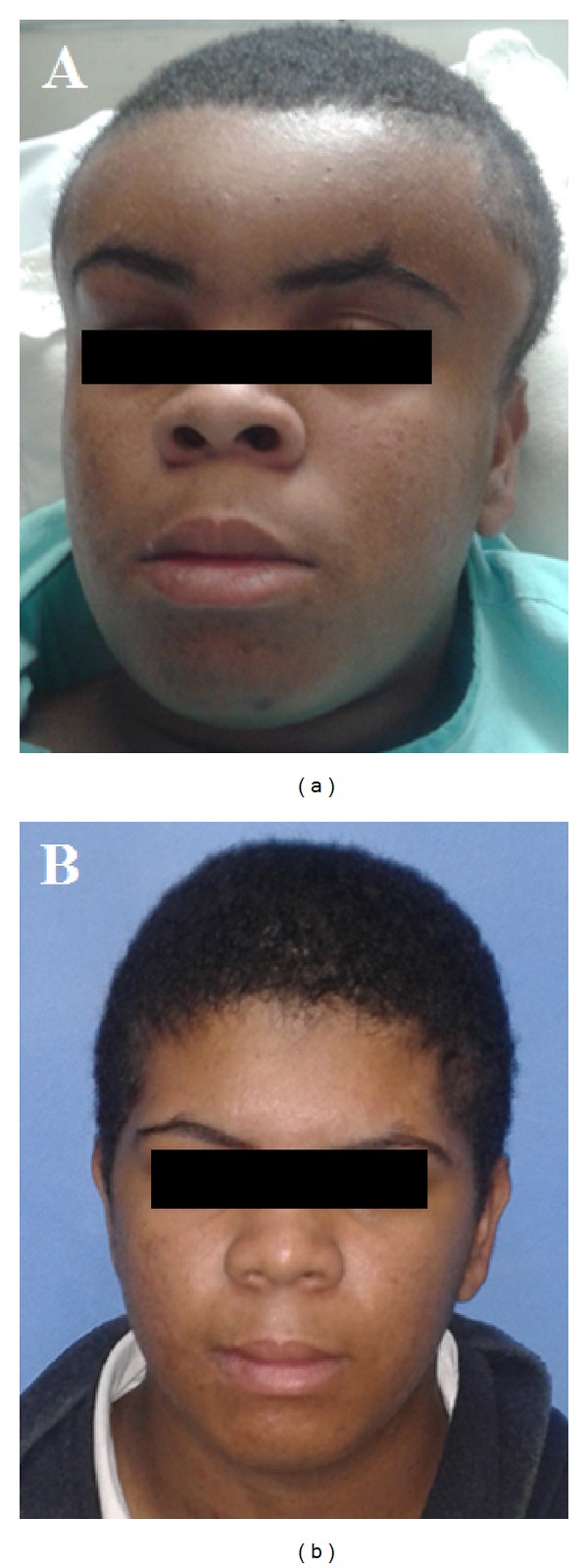
Preoperative image of the patient showing extensive forehead swelling (a), 40th postoperative day (b).

**Figure 3:**
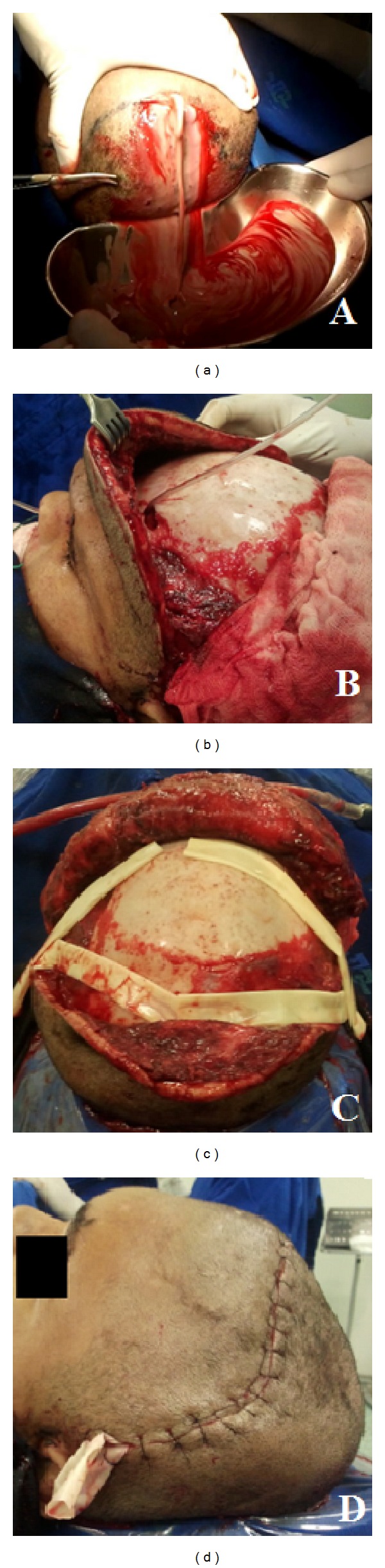
Intraoperative images. Thick purulent secretion (a). Erosion of the outer table of the frontal bone, necrosis of the temporal muscle and frontal sinus combined approach surgery (b). Penroses drains placed (c). Final appearance after suture of bicoronal incision (d).

The patient was admitted to the intensive care unit and recovered well postoperatively, afebrile, and with remission of complaints. Culture grew* Streptococcus constellatus* susceptible to multiple antibiotics. He was prescribed intravenous antibiotic therapy throughout hospitalization, treated with metronidazole 2 g/day for five days associated with vancomycin 2 g/day and ceftazidime 6 g/day maintained for 18 days. The combination of antibiotics was initially chosen guided by common microorganisms associated with this entity and adjusted according to results of antibiogram, with suspension of metronidazole. Due to suspicion of adverse drug reactions, the antibiotics were exchanged for ampicillin-sulbactam, maintained for seven more days. He was discharged on the 25th postoperative day asymptomatic and with normal physical examination, showing no new complaints in the follow-up so far (Figure 2(b)).

## 3. Discussion

Percival Pott was the first to describe Pott's puffy tumor as an indolent and circumscribed tumor of the scalp with spontaneous separation of the pericranium from the skull under such tumor [2]. It mainly affects children and adolescents, since the frontal sinus pneumatization occurs at six years old, reaching adult setting around 15 years. This allows the sinus mucosa communication with the trabecular bone by the local venous system, which favors the development of osteomyelitis [4].

Although initially described by Pott as a complication of trauma, this pathological process is developed most commonly as a complication of frontal sinusitis, with reports of other mechanisms such as insect bite [2, 3].

As this is a rare condition, it is usually diagnosed late, which favors the development of complications. Intracranial complications are observed in 60–100% of patients. Among these, there are reports of intracranial abscess, subdural and epidural empyema, cavernous sinus thrombosis and meningitis [1, 3, 5]. In the case reported, the patient was admitted to our service with an advanced history, receiving inadequate treatment, which led to the formation of extensive frontoparietal subcutaneous abscess.

In most cases, the clinical presentation consists of frontal headache, periorbital edema, purulent rhinorrhea, fever, and vomiting; all of these are reported by our patient [2, 3]. The admission physical examination, however, did not show the classic swelling of the forehead due to inadequate treatment of the disease. When the clinical presentation is suggestive, diagnostic confirmation is made by radiological exams, especially paranasal sinuses and brain computed tomography.

Culture is important to isolate the agents involved and guide appropriate antibiotic therapy, usually revealing polymicrobial involvement [1, 2]. The microorganisms commonly isolated in culture in Pott's puffy tumor are* Staphylococcus aureus*,* Streptococcus,* and anaerobes, with rare involvement of* Proteus, Fusobacterium, Bacteroides,* and* Pseudomonas* [2, 3, 5]. In our case, the culture grew* Streptococcus constellatus*, a commensal microorganism present in oropharyngeal flora and gastrointestinal and genitourinary tracts.

The management of Pott's puffy tumor requires intravenous antibiotics, analgesia, and surgical intervention in cases of persistent or worsening symptoms. The surgical approach may be external approach, endoscopic, or combined [6]. Intravenous antibiotic therapy should usually be extended by six to eight weeks postoperatively [4].

With modernization of surgical techniques, endoscopic endonasal treatment has become an excellent option in the management of Pott's puffy tumor, although there are few cases reported in the literature and the anatomy of frontal recess is complex [5]. However, because the patient presented with extensive subcutaneous collection, the most appropriate approach for the case in question was external approach combined with endonasal endoscopic surgery.

## 4. Final Comments

We present a case of Pott's puffy tumor associated with extensive frontoparietal abscess. Although considered a rare entity with the advent of antibiotics, there must be a high suspicion of this complication. Early diagnosis and aggressive medical and surgical approach are essential for a good outcome.
